# The beneficial effects of physical exercise on visuospatial working memory in preadolescent children

**DOI:** 10.3934/Neuroscience.2021026

**Published:** 2021-09-02

**Authors:** Laura Serra, Sara Raimondi, Carlotta di Domenico, Silvia Maffei, Anna Lardone, Marianna Liparoti, Pierpaolo Sorrentino, Carlo Caltagirone, Laura Petrosini, Laura Mandolesi

**Affiliations:** 1 Neuroimaging Laboratory, Fondazione Santa Lucia, IRCCS, Rome, Italy; 2 Department of Motor Sciences and Wellness, University “Parthenope”, Naples, Italy; 3 Institute de Neurosciences Des Systèmes, Aix-Marseille University, Marseille, France Department of Engineering, University “Parthenope”, Naples, Italy; 4 Department of Clinical and Behavioural Neurology, Fondazione Santa Lucia, IRCCS, Rome, Italy.; 5 Laboratory of Experimental and Behavioural Neurophysiology, Fondazione Santa Lucia, IRCCS, Rome, Italy; 6 Department of Humanities, University of Naples Federico II, Naples, Italy

**Keywords:** physical activity, sport, radial arm maze, ecological task, cognition, active lifestyle

## Abstract

The relationship between physical exercise and improvement in specific cognitive domains in children and adolescents who play sport has been recently reported, although the effects on visuospatial abilities have not yet been well explored. This study is aimed at evaluating in school-age children practicing artistic gymnastics the visuospatial memory by using a table version of the Radial Arm Maze (table-RAM) and comparing their performances with those ones who do not play any sport. The visuospatial performances of 14 preadolescent girls practicing artistic gymnastics aged between 7 and 10 years and those of 14 preadolescent girls not playing any sport were evaluated in the table-RAM forced-choice paradigm that allows disentangling short-term memory from working memory abilities. Data showed that the gymnasts obtained better performances than control group mainly in the parameters evaluating working memory abilities, such as within-phase errors and spatial span. Our findings emphasizing the role of physical activity on cognitive performances impel to promote physical exercise in educational and recreational contexts as well as to analyse the impact of other sports besides gymnastics on cognitive functioning.

## 1. Introduction

Clinical and experimental evidence shows that physical exercise (PE) is able to cause structural and functional brain changes [1]–[3]. These modifications affect the processes of brain functioning and psychological well-being [4],[5]. The multiple benefits observed in individuals who practice PE have contributed to deepen and characterize these effects. According to current knowledge, PE is a protective factor for different pathologies and mental disorders, such as anxiety and depression and it is an effective weapon to counteract physiological and pathological aging [6],[7]. Many studies have focused on understanding the biological mechanisms that regulate the phenomena of neuroplasticity induced by PE [5], as well as on correlating PE and improvement of cognitive functions [8]. While in adults these issues have been extensively investigated and the ongoing research is in an attempt to understand which type of PE is needed to produce beneficial effects (i.e. aerobic *vs*. anaerobic) [9]–[11], the studies on children and adolescents are not well deepened. In fact, the few studies performed on children and adolescents mainly concern the effects of PE in relation to academic successes [12],[13]. This approach probably depends on the difficulty in comparing data obtained in children performing PE with those obtained in sedentary children, because typically developing children spontaneously move, run, play, and do not stand still. Therefore, selecting a “real” control group becomes difficult. However, evidence show that children with higher levels of PE display best performances in learning and memory tasks in comparison to children who do not play any sport [13]–[15]. Interestingly, these beneficial effects are associated with larger size of the hippocampal regions and increased volume of cortical areas involved in action planning and decision-making processes [16]. Furthermore, positive effects of PE correlated to academic achievement have been observed on attention, working memory [17]–[21], and flexible modulation of cognitive control processes [22]–[24]. Recently, it has been reported that competitive young athletes display better performances in mental imagery skills in comparison to non-athletes ones [25]. These and other evidence suggest that the sport trains all brain processes. In fact, each action is cognition (i.e. elaboration, planning, choice of strategy, decision making). Therefore, any type of PE (or sport) is able at exercising brain functioning determining improvements in behaviours [5]. Then, PE allows the development of many cognitive domains and for this reason it is a fundamental factor for growth.

Although evidence has documented that PE is an effective tool for improving academic achievements, cognitive abilities, and emotion regulation [21],[26], the findings are rather heterogeneous both for type of PE analyzed and assortment of cognitive assessment [13].

Like other cognitive domains already investigated in relation to PE, such as executive function, also the spatial cognition appears to be crucial in growth. In particular, to explore any environment, it is necessary to know *what* environmental elements are present, *where* they are positioned [27]–[29], and *how* to implement strategies to reach (or avoid) them [30],[31]. These components, commonly defined “declarative knowledge” and “procedural knowledge” [32],[33], are fundamental in everyday life because both of them allow to orient oneself, move from place to place, form a cognitive map of environments and places, and interact with others [34]. Moreover, the exploration of an environment implies putting into action specific navigational strategies (praxis, taxis, place) that generally develop during the first decade of life [29],[35]. In addition, spatial abilities become essential also for the development of other cognitive domains. For example, closely linked to them are the attentional processes: to move it is necessary to direct the attention towards objects to be reached or obstacles to be avoid [29]. Moreover, proper functioning of spatial abilities becomes a prerequisite also for the socialization and, last but not least, to achieve autonomy. Although PE is closely related to spatial exploration and to the other cognitive domains, there is still no evidence that PE may affect spatial knowledge in children and adolescents, as it does in adults and especially in the elderly. Understanding how much PE is able to develop spatial abilities it could be useful to promote physical education in schools and a lifestyle based on PE.

The objective of the present study was to evaluate the capacities of short-term spatial memory in children practicing artistic gymnastics and to compare their performances with those of children practicing no sport. As PE we chose the artistic gymnastics because it involves aerobic and anaerobic components, requires a combination of explosive muscle contractions, elicits high heart rate [36]. In addition to these characteristics, artistic gymnastics trains all body muscles and develops strength, coordination, balance, rhythm, speed, appearing thus a complex and complete PE to be correlated to orientation spatial processes. It is important to underline as some of artistic gymnastics characteristics are very important for cognition. Indeed, while aerobic activity stimulates blood circulation in the neural circuits (i.e the prefrontal circuits are correlated to visuospatial working memory) [5],[9],[37], the coordination, the rhythm and the speed allow to move efficiently in an environment to explore it comprehensively and with little effort.

In the present study, we have chosen to study a specific aspect of spatial abilities that is visuospatial memory easily investigated with the methodological tools present in the literature. In particular, to evaluate visuospatial abilities, we used the table version of the Radial Arm Maze (table-RAM) [29], a task that reproduces in small scale the classical walking RAM task [30],[31],[35]. While the walking RAM task allows to separately evaluate declarative from procedural components, and to study allocentric memory processes, the table-RAM allows to evaluate visuospatial information processing correlated to peripersonal space [29]. In accordance with our previous studies [29]–[31], we administered the forced-choice RAM paradigm that allow evaluating short-term visuospatial memory disentangling short-term memory from working memory abilities.

## 2. Materials and methods

### 2.1. Participants

Fourteen preadolescent girls practicing artistic gymnastics (GYM group) and 14 preadolescent girls who did not practice any sport (no-GYM group) were recruited to participate in the current study. All participants were aged between 7 and 10 years (mean chronological age: 8.21 years ± 1.1) and were right-handed and native Italian speakers (Table 1). There was no significant difference in the mean age between groups (F_1,26_ = 0.28, p = 0.87).

**Table 1.** Demographic characteristics of the sample.

age	GYM group	No-GYM group
7	N = 3	N = 3
8	N = 6	N = 6
9	N = 4	N = 4
10	N = 1	N = 1
Total	N = 14	N = 14

In the present study, only children found to be behaviourally normotypic on the psychological evaluation were considered. Participants belonging to GYM group had a minimum of 2 years of training in artistic gymnastics with a frequency of three times a week, while participants belonging to no-GYM group had no continuous experience in any sport. No-GYM group was formed by children of different classes of the same primary school attended by participants of GYM group. As a criterion for the inclusion in the no-GYM group, the negative response of their parents to the question “Has your daughter ever played sports after school?” was considered.

BVN 5–11 neuropsychological battery [38] was administered only to GYM group in order to evaluate the potential correlations between the benefits induced by artistic gymnastics and cognitive performance.

Written informed consent was obtained from all the parents of the participants. The study had been approved by the Local Ethics Committee of Fondazione Santa Lucia, Rome, Italy and was carried out in accordance with the declaration of Helsinki.

### 2.2. Table radial arm maze (table-RAM)

**Figure 1.**
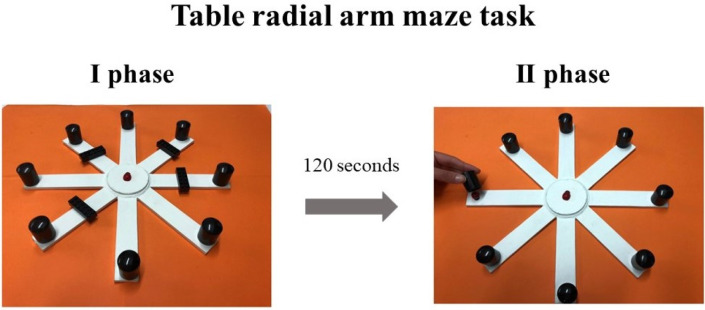
Views of the table Radial Arm Maze (RAM). The figure shows the two phases of the forced-choice paradigm of the task. See text for further details.

The table-RAM is made of a round central platform (5 cm in diameter). Eight white arms (3 cm wide × 25 cm long) depart the central platform like the spokes of a wheel. At the end of each arm, a small black round cap (2.5 cm in diameter × 2.8 cm height) covered the reward (a little coloured wooden ladybug). As detailed in the next section, in the first phase of the task, four arms of the maze were closed by pieces of black plastic (3.5 cm wide × 1 cm long × 1 cm height) (Figure 1). The table-RAM was placed on an orange desk. All extra-maze cues (windows, paintings, posters, doors, and experimenter) were held in constant spatial relations throughout the experiment. The RAM arms were virtually numbered clockwise, arm 1 being in front of the children. Participants had visual access to the table-RAM only during the experiment.

### 2.3. Experimental procedure

In according to Foti and colleagues [29], the table-RAM task was presented as “Ladybug game”. The child had to move the older sister ladybug (“Ladybug”) which was placed on the central platform, to find its sisters hidden inside the caps at the end of each arm of the RAM. To increase participants' motivation, at the end of each trial the child received a reward (a coin) in exchange for the ladybugs discovered. All children performed the table-RAM forced-choice paradigm after a short training (3 trials) of habituation to the task in the classic free-choice paradigm. In this training phase, the child could explore the eight arms freely to find the ladybugs hidden inside the caps at the ends of each arm.

After the habituation training, all children were tested in the forced-choice paradigm consisting of two phases. In the first phase, although all arms contained the ladybugs on them, only four arms (for example, arms 1, 3, 4, 7) were accessible, because the remaining four arms were closed at the proximal end of each arm (Figure 1). Different angles separated the open arms to avoid the children reaching the solution through the employment of a procedural strategy (as occurring exploring for example arms 1, 2, 3, 4 or 2, 4, 6, 8). The task started with the Ladybug placed on the central area of the maze and the participant was allowed to explore the four open arms by moving the Ladybug to collect the four accessible ladybugs. Afterwards, each child was invited to interrupt the game for 120 s before the second phase of the task started. During this time, the child was kept in a different place and invited to chat with the experimenter. In the second phase, the participant was allowed moving the Ladybug in all arms, but only the four previously closed arms were rewarded (since the other four ladybugs had been collected in the previous phase). The arrangement of the table-RAM and the child's position was identical to the first phase. The successes in visiting only the rewarded arms essentially depended on remembering which arms had already been visited, stressing the memory component and neglecting the search patterns. In according to procedures describes by Foti and colleagues [29], each participant performed three trials a day for two consecutive days (two sessions), with an inter-trial interval of at least 1 h. In each of the six trials, a different configuration of closed arms was used. At the beginning of the first trial, the experimenter explained the task to each participant using the same simple verbal instructions (“Do you remember the Ladybug that had to find its sisters under the black upturned caps? Well, the mischievous sisters have hidden themselves again. However, they do not know you're there to help the Ladybug find them. Now, some corridors are blocked. You have to let the Ladybug enter only in the arms that are open. Go!”). The verbal instructions after the 120 s interval were: “Uh! Something now has changed, there are no blocks anymore. Then, the Ladybird can freely go and look for the other sisters! Good job!”).

The parameters considered in the second phase were the *entries*, defined as the total number of the visited arms (correct and incorrect); *spatial span*, defined as the longest sequence of correctly visited arms (ranged from 1 to 4); *short-term memory errors*, defined as the re-visits into already visited arms. This parameter was broken down further into two error subtypes: *across-phase errors*, defined as visits into an arm that had been visited during the first phase of the same trial; *within-phase errors*, defined as re-visits into an arm already visited in the same phase. While the across-phase errors are correlated to short-term memory, the within-phase errors to working memory. Briefly for each table-RAM parameter (entries, spatial span, short-term memory errors) obtained during task, the average of the correct responses and errors were computed for each participant.

The choices made by the participants during the forced-choice task were videotaped and registered manually.

### 2.4. Statistical analyses

All data were presented as the mean ± SD and were first tested for normality (Shapiro-Wilk's test) and homoscedasticity (Levene's test). When normally distributed, data were analyzed by using one-way ANCOVA, the age was entered as covariate of no interest (i.e. entries, spatial-span and short-term errors). When data were not normally distributed, non-parametric analyses (Mann-Whitney U, Wilcoxon's test) were used (i.e., across-phase errors and within-phase errors). The two-tailed Pearson's correlation analyses were used to test for correlations between the score of the BVN 5–11 and years of gymnastics practiced by girls of the GYM group. Moreover, correlation analyses were made between the results of the table-RAM forced-choice task and the ones of the BVN 5–11.

Statistical analyses were performed using SPSS version 23 Statistical Software Package for Windows (SPSS Inc., Chicago, IL, United States). The significance level was defined as p < 0.05.

## 3. Results

All children completed the first phase of table-RAM task without errors. In fact, no one entered an arm already visited, and all children collected the four ladybugs. In the second phase, when all the arms were opened and the participants could move the Ladybug without restrictions, significant statistical differences between groups were found.

### 3.1. Entries

**Figure 2.**
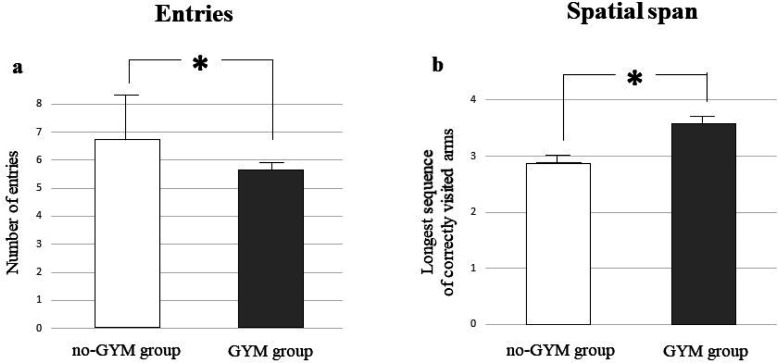
Performances of GYM group in comparison to no-GYM group in the second phase of forced choice paradigm. Data are expressed as mean ± SD. The asterisks indicate the significance level of ANCOVAs (∗ p < 0.05). See text for further details.

A one-way ANCOVA on number of entries, a parameter expressing the general performance level, revealed that children practicing artistic gymnastics made a significantly lower number of entries than children who did not practice sport (GYM group: *x* = 5.3 ± 0.64; no-GYM group: *x* = 6.7 ± 2.3; F_(1, 26)_ = 4.88, p = 0.04, η_P_^2^ = 0.16) (Figure 2a).

### 3.2. Spatial span

A one-way ANCOVA on the longest sequence of correctly visited arms in the second phase of the task revealed that children practicing artistic gymnastics had a significantly higher spatial span value than children who did not practice sport (GYM group: *x* = 3.6 ± 0.7; no-GYM group: *x* = 2.8 ± 0.6; F_(1, 26)_ = 6.2, p = 0.019, η_P_^2^ = 0.19) (Figure 2b).

### 3.3. Short-term memory errors

**Figure 3.**
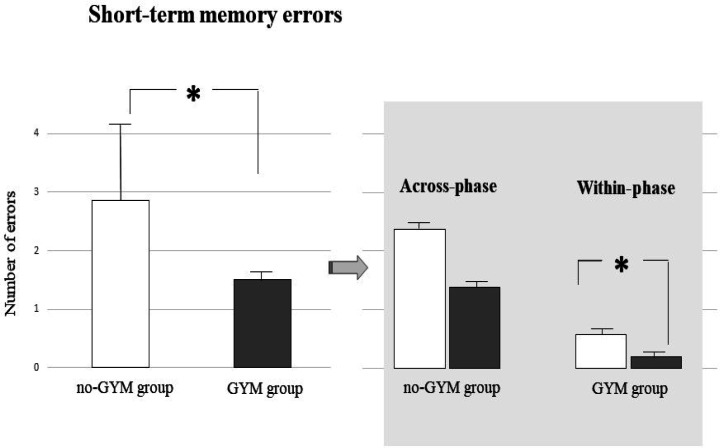
Short-term memory errors divided in across- and within-phase errors performed by the two groups of children in the second phase of the forced-choice paradigm. Data are expressed as mean ± SD. The asterisks indicate the significance level of ANCOVA (∗ p < 0.05) (left side) and the significance level of Mann-Whitney U (∗ p < 0.05) (right side). See text for further details.

A one-way ANCOVA on short-term memory errors performed in the second phase of the task revealed that children who did not practice sport made a significantly higher number of short-term memory errors than children practicing artistic gymnastics (GYM group: *x* = 1.4 ± 0.6; no-GYM group: *x*: 2.8 ± 2.25; F_(1, 26)_ = 4.88, p = 0.04, η_P_^2^ = 0.16) (Figure 3). To better understand if the significant reduction of errors by the gymnasts was due to a superior functioning of the short-term or working memory processes, we analyzed the two subtypes of short-term memory errors: across-phase errors (visits into an arm that had been entered during the first phase) and within-phase errors (re-visits into an arm previously visited in the same phase). While across-phase errors reflect a short-term memory deficit, within-phase errors express a working memory deficit. Non-parametric analyses (Mann-Whitney) revealed that no-GYM group made a significantly higher number of within-phase errors compared with GYM group (GYM group: *x* = 0.1 ± 0.2; no-GYM group: *x*: 0.5 ± 0.6; U = 57, Z = −2.07, p = 0.04). A similar pattern was observed in relation to across-phase errors, even if the statistical difference did not reach the significant level (GYM group: *x* = 1.4 ± 0.6; no-GYM group: *x*: 2.2 ± 1.7; U = 64, Z = −1.6, p = 0.057). When considering the distribution of errors between groups, we observed a significant lower number of errors in the within phase in the GYM group in comparison to NO-GYM one (Chi^2^ = 3.6, d.f = 1, p = 0.05).

### 3.4. Analysis of correlation

**Figure 4.**
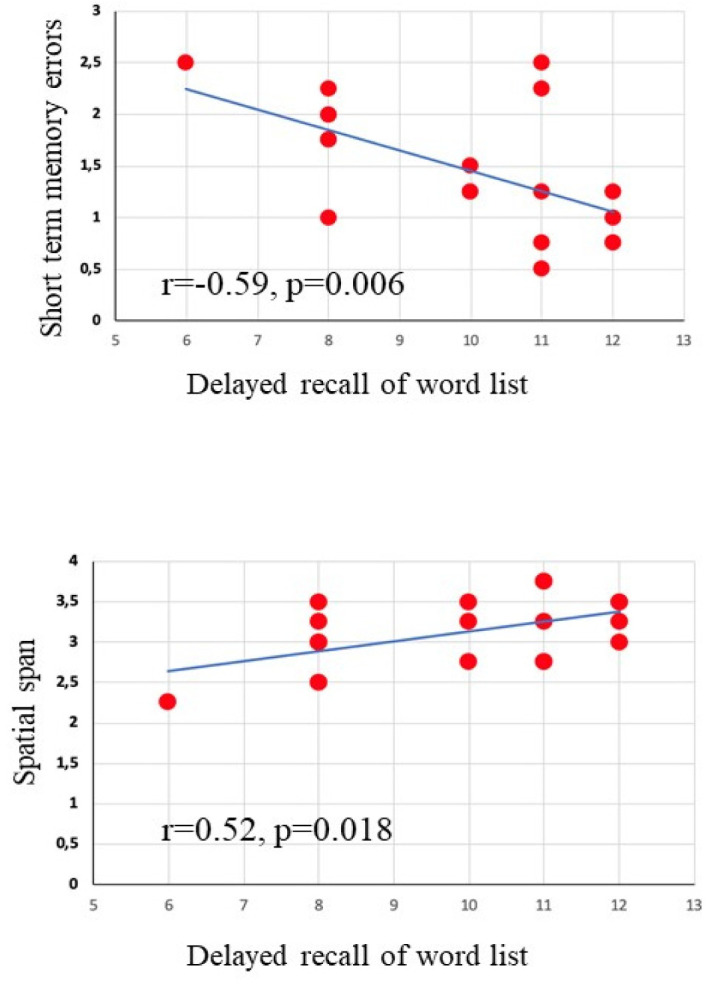
Correlations between the subtest delayed recall of word list and two parameters of table-RAM forced choice task (short term memory errors and spatial span).

In Table 2, the two-tailed Pearson's correlations analysis between the neuropsychological scores of BVN 5–11 and years of gymnastics practiced by girls of the GYM group are shown, while in Figure 4 the significant correlations between the subtest delayed recall of word list and two parameters of table-RAM forced choice task (short term memory errors and spatial span) are reported. No significant correlations resulted when considering the other scores of BVN 5–11 and the parameters of the RAM.

## 4. Discussion and conclusions

The present study analyzed in school-age children the effects of physical exercise (PE) on short-term visuospatial memory by means of a table version of the RAM [29] that reproduces in small scale the walking RAM task [30],[31],[35]. In particular, we focused on forced-choice paradigm of the RAM that allows analysing different facets of the short-term memory processes. In fact, the two subtypes of short-term memory errors, *across-phase errors* (visits into an arm that had been entered during the first phase) and *within-phase errors* (re-visits into an arm previously visited in the same phase), permit to differentiate short-term components (across-phase errors) from working memory ones (within-phase errors).

**Table 2.** Correlations derived from BVN 5–11 and years of gymnastics practiced by girls of the GYM group.

	BVN 5–11 neuropsychological battery
	test	r	p
PERCEPTION	Visual discrimination (time)	−0.615	0.004
	Auditory discrimination	0.514	0.020
PRAXIS	Upper limb gestures	0.473	0.035
CALCULATION	Forward enumeration (time)	−0.484	0.031
	Total accuracy	0.462	0.046
REASONING	Raven's Progressive Matrices	0.537	0.015
VISUO-SPATIAL SHORT-TERM MEMORY	Corsi block tappingTest	0.445	0.050
VISUO-SPATIAL LONG-TERM MEMORY and PRAXIA	Rey's complex figure (copy)	0.580	0.009
	Rey's complex figure (delayed recall)	0.492	0.032
VERBAL LONG-TERM MEMORY	Delayed recall of word list	0.431	0.050
EXECUTIVE FUNCTION	Phonological verbal fluency	0.440	0.059

The main result of the present research is that PE, when regularly and consistently practiced, as is the case of the artistic gymnastics we analysed, impacts on visuospatial working memory. In particular, comparing the performances of preadolescent girls practicing artistic gymnastics (GYM group) with those who do not practice any sport (no-GYM group), we found significantly reduced the number of within-phase errors in the GYM group (Figure 3). This result agrees with previous studies showing in young populations a correlation between extended chronic exercise and improved working memory [19],[39]. Chronic exercise is a type of PE, which consists of multiple exercise sessions per week and lasts for an extended period (typically over 6 weeks). Although we cannot maintain that our gymnasts were trained multiple times per week, we can state that their PE was an extended physical activity, given that being trained for at least two years with a frequency of three times a week was an inclusion criterion in the GYM group. Therefore, even if the GYM group did not perform exercises in a strictly “chronic” way, the present findings indicate that a prolonged and intensive training is associate with an improvement in the functioning of some cognitive domains, such as spatial working memory abilities.

The present data closely fit with the improvement of working memory correlated to PE in a young population previously reported [19],[39] that however do not concern the spatial domain. In fact, as underlined in the Introduction, most of these studies are focused on executive domain in general (i.e., attention, working memory, inhibition) [40].

The better functioning of visuospatial working memory in gymnasts is also reflected in the other parameters, such as errors and spatial span (Figure 2a,b), suggesting thus that an efficient availability of information in the short-term memory storage allows a better resolution of the task. In support of the present data, some voxel-based morphometry studies have evidenced that the gymnasts exhibit higher grey matter volume in specific cortical areas, such as prefrontal cortices correlated to the working memory [41]–[43] emphasizing from a psychobiological perspective that PE does determine changes in the brain structure.

Interestingly, we found in the GYM group significant correlations between years of practice of gymnastics and several cognitive functions such as perception, praxis abilities, calculation, deductive logical reasoning, verbal and visuospatial long/short-term memory (Table 2). Moreover, we found significant correlations between the performances obtained at the verbal long-term memory tests and the number of RAM errors (Figure 4). Although these data are very preliminary, they indicate a relationship between PE and cognitive functioning, suggesting thus as the neuroplastic effect exerted by PE on the brain may result in enhancement of cognitive functions. However, these findings shall be considered with cautions and further investigations will be needed.

The present study does not allow to establish whether the better performances of gymnasts in working memory abilities depend on the specific type of physical training. In other words, is it due to gymnastics if spatial working memory works better? Certainly, the artistic gymnastics facilitates abilities, such as the estimation of the direction of movements, the speed of execution, and the identification of their own and surrounding objects' locations [44], but these considerations cannot be sufficient to definitely answer the question. Other studies using the table-RAM should be performed on other young people playing sports, for example considering open skill sport, that take place in an unpredictable and constantly changing environment where movements need to be continually adapted, such as basketball, soccer [45]. In addition to this question, it is necessary to underline the limitations of the present study which cannot be overcome requiring caution when generalize the data. In particular, the small size of the samples and the inclusion of only female participants do not allow to generalize completely the results and replicate the study. Another limitation concerns the age of the participants (Table 1) which ranged from 7 to 10 years. We know that spatial abilities are not fully developed in children younger than about 10 years of age [30],[35],[46] and this could determine variability. However, these limitations can be overcome by increasing the sample in future studies. Moreover, further studies based on randomized controlled designs are desirable to better investigate the possible causal relation between PE and the cognitive improvements.

Although these aspects deserve remarkable attention, the present results indicate that the constant practice of the artistic gymnastics may contribute to improve spatial working memory, suggesting thus specific promotion of this type of PE in educational contexts. In this line, it is important underline that it has been observed that the spatial aspects of working memory are crucial to arithmetic learning and problem solving [47]. Therefore, promoting specific exercises capable of enhancing visuospatial skills could be a targeted and effective educational strategy. Unfortunately, PE is limited in Italy but we hope for the present results and further researches may shed light on the importance of PE in improving cognitive functioning during childhood-adolescence. Moreover, we wish for these evidences lead to enhancement of school programs in Italy and other countries where PE is more limited.
